# Adaptive Team Performance: The Influence of Membership Fluidity on Shared Team Cognition

**DOI:** 10.3389/fpsyg.2019.02266

**Published:** 2019-10-09

**Authors:** Wendy L. Bedwell

**Affiliations:** Fogelman College of Business and Economics, University of Memphis, Memphis, TN, United States

**Keywords:** team adaptation, adaptive team performance, team composition, dynamic team, team membership change, membership fluidity, team mental models, team cognition

## Abstract

Team membership change literature has traditionally focused on performance effects of newcomers to teams. Yet, in practice, teams frequently experience membership loss *without* replacement (e.g., downsizing) or membership exchanges—replacing a member who has left the organization with a current, experienced employee. Despite the prevalence of these practices, little is known about the impact of such changes on team performance. Drawing upon two complementary team adaptation theories, the influence of both membership loss without replacement and loss with replacement by experienced personnel on the cognitive processes underlying adaptation (operationalized as development of effective team mental models – TMMs) was examined. Results suggested that Teammate TMMs (i.e., shared knowledge of member preferences/tendencies) and Team Interaction TMMs (i.e., shared knowledge of roles/responsibilities) are differentially influenced by the movement of members in and out of teams and differentially predict adaptive team performance. Further, TMM measurement choice (i.e., the use of similarity versus distance scores) matters as relationships differed depending on which metric was used. These results are discussed in the context of team adaptation theory, with implications for strategic human resource management.

## Introduction

Downsizing has become common for organizational survival, as evidenced by the 2009 economic recession, when mass layoffs (i.e., ≥50 employees) increased dramatically (US Department of Labor Bureau of Labor Statistics, 2011). In work teams, downsizing creates membership loss without replacement *or* requires job rotation of current employees into new teams; here these “new members” are not novices but have task experience. Despite the prevalence of such practices, little is known about their impact, as research has rarely compared dynamic to stable team configurations, let alone membership loss to membership replacement (Tannenbaum et al., 2012).

With the exception of work on team downsizing (DeRue et al., 2008), research on membership fluidity—the dynamic flow of members in and out of teams (e.g., Edmondson et al., 2001; Edmondson, 2003; Tannenbaum et al., 2012)—has historically focused on newcomer socialization (see Moreland and Levine, 2001 for a comprehensive review). However, organizational performance outcomes largely depend on the ability of teams to quickly adapt their processes to rapidly changing demands (Burke et al., 2006), such as varying membership (e.g., Bedwell et al., 2012). Thus, such research is important.

Surprisingly, the underlying *cognitive processes* of adaptation in teams experiencing membership change have also received little attention in the team adaptation research, despite the prevalence of “learning” and “team cognition” constructs in prominent theories focusing on how teams adapt to change. One particular cognitive process often associated with effective team adaptation is the development and/or change of team mental models (TMMs), which are organized knowledge structures shared among members of a team (Cannon-Bowers et al., 1993; Mathieu et al., 2000). The two prevailing models of adaptation in the literature, Kozlowski et al. (1999) and Burke et al. (2006), highlight the importance of these cognitive structures. Burke and colleagues include TMMs within the learning phase of their multiphasic model of team adaptation. Kozlowski et al. (1999) did not specifically mention TMMs in their theory of adaptive teams; yet, they did argue for the importance of developing *shared knowledge* regarding tasks, team roles, role boundaries, and other team members—which is the definition of the various TMMs originally outlined by Cannon-Bowers et al. (1993). Both theories suggest that increasing sharedness of TMMs regarding both task and team members *should* enable teams to adapt to any number of situations (Kozlowski et al., 1999; Burke et al., 2006).

Thus, this effort seeks to advance the team adaptation literature by testing the effects of membership change on performance via development of shared TMMs. The contribution is twofold: (1) integrating two complementary models of team adaptation (Kozlowski et al., 1999; Burke et al., 2006) and (2) offering the first empirical test of multiple membership change types (i.e., loss and exchange) against stable teams, thereby addressing the call by Tannenbaum et al. (2012) for simultaneous investigations into various member change configurations.

### Membership Change

Membership change has two main schools of thought. On one hand, some defend membership change, suggesting it can increase the available cognitive resources of a team (Kane et al., 2005) and fuel reflection on team processes (Sutton and Louis, 1987; Feldman, 1994). Researchers argue that such activities enable members to draw from a broader knowledge base, develop greater shared thinking regarding how the team should continue to operate and, ultimately, improve performance outcomes (Ancona, 1990; Gersick and Hackman, 1990; Waller, 1999).

A second school of thought, however, suggests that membership change is detrimental to team performance. Members take knowledge with them when they leave (Cascio, 1999), which eliminates access to that individually held knowledge (Argote, 1999). In tasks where performance hinges on the ability of members to pool relevant knowledge, loss of a member (and thereby, loss of knowledge) can lead to performance decrements. With regard to membership replacement or loss, research has found that after a member change, attention is temporarily diverted from the task because teams are in a state of flux (i.e., dynamic, unstable interaction pattern; Summers et al., 2012). Essentially, when teams take time away from a task (e.g., for socialization of a new member), they face potential process loss (Steiner, 1972).

Additionally, stable membership leads to teammate familiarity, which has been linked to positive outcomes such as cohesion, coordination, low anxiety, willingness to express disagreement, and performance, in both lab and field studies (e.g., Levine and Moreland, 1991; Gruenfeld et al., 1996; Kim, 1997; Moreland et al., 1998). Although some studies have found familiarity to have negative or curvilinear effects (e.g., Katz, 1982; Berman et al., 2002; Sieweke and Zhao, 2015), any positive benefits are certainly not afforded to teams with new members (i.e., membership replacement). As the task in the present study required effective pooling of distributed information, in accordance with the second school of thought, it is hypothesized that teams experiencing membership loss or replacement would experience performance decrements as compared to teams with stable membership.

Hypothesis 1a and b: (a) Membership loss and (b) membership loss w/replacement teams will experience performance decrements as compared to intact teams.

### TMMs and Adaptive Performance

As noted above, current team adaptation theory has noted that effective adaptive processes are predicated on successful team learning, including development of shared knowledge structures (Kozlowski et al., 1999; Burke et al., 2006, 2008). Cannon-Bowers et al. (1993) have argued for the existence of several types of TMM when teams are engaged in complex tasks. They specifically addressed four types. Team members must have a shared understanding of the *technology/equipment* required for task completion. Members must also share knowledge structures regarding the *task*, specifically procedures, task strategies, constraints and resources. Third, teams share knowledge regarding *team interaction*, which is comprised of the roles/responsibilities, interaction patterns, interdependencies, and information flow. Finally, teams can have shared knowledge regarding members of the *team* itself, including knowing other members’ skills, attitudes, preferences and tendencies.

Mathieu et al. (2000) considered the difficulty in operationalizing these four types within a single study and suggested all four types essentially depict two major content domains: team relevant information and task relevant information. Arguably, collapsing the Task TMMs does make sense in this effort as it is difficult to separate the components of those two dimensions (e.g., there is no specialized equipment therefore knowing the operating procedures naturally involve knowing the task procedures). However, maintaining distinction among the Team Interaction and Team TMMs is important in this particular study, as members can have a shared understanding of the roles/responsibilities and interaction patterns (i.e., Team Interaction TMMs) without having a shared understanding of members preferences (i.e., Team TMMs).

#### Task TMMs

When teams experience replacement of a member with a task-experienced one, task knowledge (e.g., task procedures, strategies, resources, and operating procedures) can remain highly shared when information is standardized. However, even in the most standardized tasks, team members bring their own task conceptualizations and views regarding appropriate task strategies (Burtscher and Manser, 2012). Thus, in teams with membership replacement, new members may have different task conceptualizations. Alternatively, when there is membership loss without replacement, teams must reconfigure. This can require changes in task conceptualizations, which can negatively influence sharedness when teams are under time pressures and unable to articulate new views (Rico et al., 2008). Also, if there are different ways to achieve effectiveness (as is the case in this study), this can further inhibit sharedness, as evidenced in the difficulty of short-lived (Rico et al., 2008) and *ad hoc* fluid (Kolbe et al., 2009) teams in developing shared cognition.

Team mental models sharedness is positively related to performance (DeChurch and Mesmer-Magnus, 2010a, b) and it is anticipated that these findings will also extend to adaptive performance. Indeed, research on Task TMMs and adaptive performance suggests that Task TMMs aid adaptive performance in novel environments (Waller et al., 2004). However, TMMs are only one aspect of teamwork (e.g., attitudes, behaviors, and cognitions; Salas et al., 2009), and therefore, a team’s composition can influence team performance through a variety of mediators beyond shared cognition (see Mathieu et al., 2008). Given this complex relationship, partial mediation is hypothesized:

Hypothesis 2: Task TMMs will partially mediate the relationship between membership fluidity and performance, with intact teams developing more similar Task TMMs than membership loss and replacement teams.

Team Interaction TMMs are comprised of team-relevant knowledge, such as individual roles and interdependencies, interaction patterns, and information flow. It may seem as though teams experiencing member replacement with a role-experienced member will have little (or no) disruptions in development of Team Interaction TMMs (similar to intact teams) since interdependencies associated with roles/responsibilities are dictated by the task (and not specific team members). However, teams rapidly develop stable patterns of working (e.g., Gersick and Hackman, 1990; Zijlstra et al., 2012) and given that there was no “one correct” way to interact in this task for effectiveness, each team could have developed different, yet effective, interaction patterns. Thus, a member coming to a new team may have had different interaction norms than the new team and membership loss with replacement teams may show decrements in sharedness of their Team Interaction TMMs. Similarly, yet more pronounced, teams experiencing membership loss *must* redefine roles by redistributing task requirements, which can affect interdependencies. Teams failing to develop a new shared understanding of these redistributions will show decrements in Team Interaction TMMs as compared to intact teams.

Just as Task TMMs are important for team performance, it is suggested that Team Interaction TMM will also be positively related to adaptive performance. Although there is a lack of studies examining TMMs in adaptive contexts, Marks et al. (2000) found that such TMMs were stronger predictors of performance in novel, as compared to routine, environments. This supports the notion that teams with highly shared Team Interaction TMMs adapt better than teams without highly shared TMMs. This effort sought to replicate those findings in the adaptive performance context, again, arguing for partial mediation.

Hypothesis 3: Team Interaction TMMs will partially mediate the relationship between membership fluidity and performance gains, with intact teams developing more similar Team Interaction TMMs than membership loss or replacement teams.

Team mental model theory posits that team members who work together gain knowledge about each other and, thus, develop shared knowledge regarding each other’s working preferences (i.e., specific Teammate TMMs; Cannon-Bowers et al., 1993). Only a few studies have empirically investigated relationships between shared Teammate TMMs and performance (e.g., Smith-Jentsch et al., 2009). One study considered task changes and team familiarity, finding an interaction between diverse experiences and team familiarity that led to performance improvements (Huckman and Staats, 2011). This suggests that teams who know each other’s expertise and ways of working are able to overcome task changes. Such findings should also hold true for membership loss because the content of the team-specific knowledge regarding member preferences should remain relatively constant. In other words, remaining members should maintain shared understanding of other’s preferences, knowledge, attitudes, regardless of who remains on the team as membership does not dictate how people approach their work. In contrast, membership replacement teams must integrate an unknown member, which should negatively influence shared knowledge of member preferences, because such learning takes time (Akgün and Lynn, 2002)—time that teams required to rapidly adapt to new members rarely have.

Teammate TMMs should be important for performance, just like Task and Team Interaction TMMs. Indeed, research has found that teammates with prior working experience showed greater agreement with respect to their Teammate TMMs, which partially explained the relationship between familiarity and the willingness to ask for/accept assistance (Smith-Jentsch et al., 2009). These findings suggest that a team’s ability to adapt (e.g., by compensating for one another) is undermined by a lack of shared Teammate TMMs. Furthermore, research has demonstrated that teams who train together perform better because they have greater knowledge of one another (Liang et al., 1995). It follows that more highly shared Teammate TMMs should enable teams to realize performance gains as compared to teams without such sharedness.

Hypothesis 4: Teammate TMMs will partially mediate the relationship between membership fluidity and performance, with intact teams developing more similar Teammate TMMs than membership replacement teams.

**FIGURE 1:**
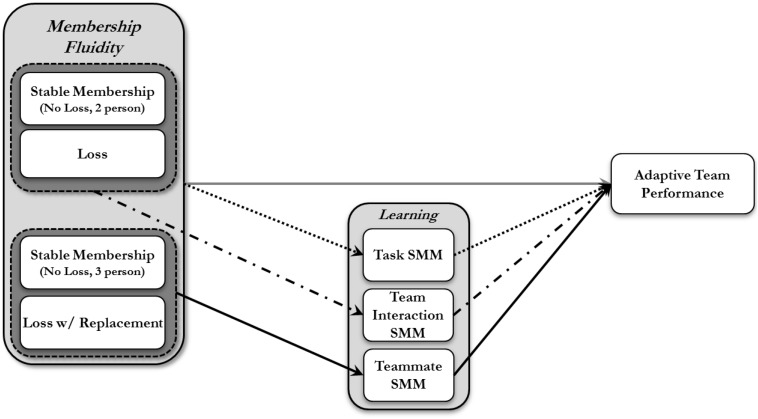
Hypothesized relationship among study variables.

Essentially, the proposed model argues that shared TMMs partially enables performance and mitigates the negative influence of membership replacement/loss on performance (see Figure 1).

## Materials and Methods

### Participants

Hundred and sixty five undergraduate and graduate students (71 males, 93 females, one declined to state gender) from a university in the southeastern U.S. were randomly assigned to 60 teams in four conditions: (a) a *two-member control* condition (15 teams, *N* = 30); (b) a *three-member control* condition (15 total teams, *N* = 45); (c) a *membership replacement* condition (i.e., where a lost team member was replaced with an experienced participant from another team; 15 teams, *N* = 45) and (d) a *membership loss* condition (i.e., loss of participant without replacement; 15 teams, *N* = 45). Two control conditions were used to avoid the confound of team size accounting for performance outcomes. Thus, membership loss teams were always compared to the two-person control team and membership exchange teams were always compared to the three-person control condition.

Participants received a cash stipend ($10/h, $25 total). To ensure high levels of motivation and encourage keeping manipulations confidential, participants were eligible to win a performance reward ($25/participant for top teams; $20 and $15/participant for 2nd and 3rd place teams, respectively). Treatment of participants was in accordance with APA ethical guidelines and federal regulations, and the study had been reviewed and approved by the university’s Institutional Review Board (IRB). Written consent was waived by the IRB as that would be the only identifiable information tying participants to the study. Consent was indicated by completion of the study as all participants were informed of their right to withdrawal at any time. No participants withdrew.

### Procedure

Teams engaged in an interactive, computer-based simulation set in an emergency room waiting area, filmed from a first-person view. Actors portrayed the role of doctors, volunteers, and patients. Participants “interacted” with the characters in the video verbally, simulating a real conversations even though it was recorded video (see Smith-Jentsch, 2007). The simulation was similar across performance periods and identical across conditions. There were three roles: *Waiting Room Staffer*, *Records Staffer*, and *Claims Staffer* (the Claims and Records roles were combined in two-person teams). The Waiting Room Staffer interacted directly with the simulation, answering patient/staff questions and responding to voicemails. The Records Staffer maintained: (a) an employee tracking form and (b) a patient log form. The Claims Staffer completed: (i) a patient insurance claim form and (ii) a complaint form for formal complaints made against employees, and received patient details from the “admittance department.”

**FIGURE 2:**
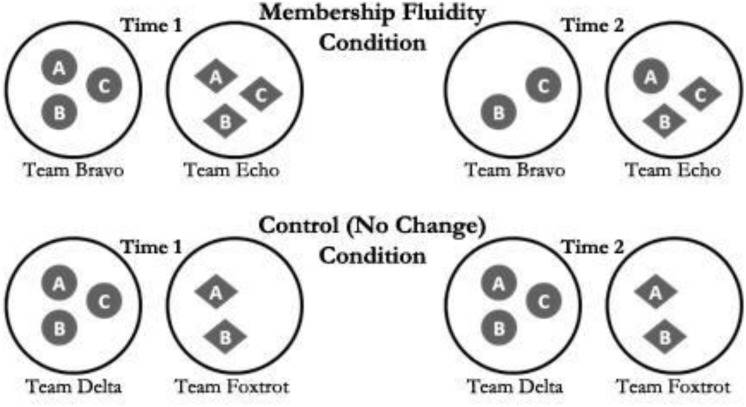
Team member configurations at Time 1 and Time 2.

Upon arrival, participants were told their purpose and that another team was working on the same simulation simultaneously. Then all members watched a training video and completed a demographic measure (e.g., age, gender, GPA, major, etc.). Using a worksheet tailored for team size, teams engaged in a 15-min planning period, performed Part I of the simulation, and then completed Time I performance measure. This was followed by the membership change event (or no change for control teams). As noted previously, there were four conditions: two-person intact teams (*Team Foxtrot*: control group with two members), three-person intact teams (*Team Delta*: control group with three members), membership loss teams (*Team Bravo:* three-person membership loss team, resulting in two remaining members), and membership replacement teams (*Team Echo:* three-person team who lost one yet gained another member, resulting in three members). After Performance Cycle I, remaining members of *Team Bravo* were told their Claims Staffer was needed elsewhere and there were no replacement personnel available (see Figure 2 for a visual representation of members across all four conditions at Time 1 and Time 2).

All teams were then told to take no more than 5 min to plan for the next phase. When finished, members completed the TMM measures; performed Part II of the simulation; completed the Time II performance measure; were debriefed, paid, and released.

### Measures

#### Demographic Information

The demographic survey included customary data such as age, gender, GPA, year in school, and major (among other data). GPA, specifically used as a covariate in this study across all analyses, was calculated as an average for the team. The mean across conditions was 2.85 (*SD* = 0.61). Skewness (−0.97) and kurtosis (0.96) levels across conditions were within acceptable ranges. The means within conditions were as follows: two-person intact teams (*M* = 3.14, *SD* = 0.45), three-person intact teams (*M* = 3.20, *SD* = 0.30), three-person membership loss teams (*M* = 3.33, *SD* = 0.42), and three-person membership loss with replacement teams (*M* = 3.23, *SD* = 0.39).

#### Familiarity

Familiarity was defined in this study as the degree to which participants knew one another. This was measured using a scale developed for use with the simulation task by Smith-Jentsch and colleagues. Familiarity was calculated as a team-level variable, averaging the level of familiarity among each dyadic pair within a team using one item – the number of months members had known one another. This was used as a control variable in analyses that considered Teammate SMMs, since greater familiarity could increase the amount of information known regarding a person’s personality characteristics. Across conditions, the mean was 4.44 (*SD* = 8.46). Within conditions, means were as follows: two-person intact teams (*M* = 1.00, *SD* = 2.36), three-person intact teams (*M* = 4.47, *SD* = 6.96), three-person membership loss teams (*M* = 4.83, *SD* = 9.04), and three-person membership loss with replacement teams (*M* = 7.45, *SD* = 11.96).

#### Role Comprehension

This original scale was designed to determine the degree to which the task training was effective. This is the only control variable measured after the initial transition phase and was used in all analyses as it directly influences Task as well as Team Interaction SMMs. Specifically, the more clarity members have regarding the roles, the better able they would be to determine what tasks are critical and how to coordinate to accomplish those tasks. The scale was either 2-items or 3-items, depending on the number of team members (2-item for two-person intact teams, 3-items for all other conditions). The items asked whether members understood the requirements of their own roles as well as the roles of the other team members. The mean across conditions was 3.73 (*SD* = 0.43). Skewness (0.31) and kurtosis (1.46) levels across conditions were within acceptable ranges. Means within conditions were as follows: two-person intact teams (*M* = 3.63, *SD* = 0.52), three-person intact teams (*M* = 3.67, *SD* = 0.41), three-person membership loss teams (*M* = 3.84, *SD* = 0.43), and three-person membership loss with replacement teams (*M* = 3.78, *SD* = 0.36).

#### Team Mental Models

Research has suggested two approaches to studying TMMs: (a) sharedness in TMMS among members, and (b) accuracy of the TMMs (i.e., the degree to which TMMs reflect an expert model). Although prior research is helpful in selecting metrics, the task often dictates their appropriateness for the measurement (Mohammed et al., 2010). In this experiment, there was no one correct way to work; therefore, interest lay in sharedness rather than accuracy. TMM sharedness was calculated as an average correlation between team members, as outlined by Smith-Jentsch et al. (2005), who argued such an approach was warranted because the indices are correlational and thus, parallel to Pathfinder C (e.g., Stout et al., 1999; Marks et al., 2002), UCFNET QAP coefficients (e.g., Mathieu et al., 2000), or coefficient alphas (e.g., Webber et al., 2000). More similar TMMs have an index closer to 1. However, sharedness indices only represent similarities in the *patterns* of responses, not the actual *closeness* of the scores. To capture this latter metric, a Euclidean distance was also calculated, where lower distance scores are indicative of closer ratings (i.e., more similar the TMMs, based on a range of 0 – 13.86).

Data for the team interaction and taskwork TMMs were captured using a structured network approach (e.g., paired comparisons), because prior research suggested it is most predictive of adaptive performance (Resick et al., 2010). Participants were given a matrix of all tasks (or relevant teamwork attributes) and instructed to rate each attribute in relation to all other attributes for that model using a scale ranging from “−4” (= *high degree of one requires low degree of the other*) through “0” (= *unrelated*) to “4” (= *high degree of one requires high degree of the other*). The ratings were completed before Performance Cycle II, yet after the membership change event (Task similarity: *M* = 0.38, *SD* = 0.24, Task distance: *M* = 12.00, *SD* = 3.92, Team Interaction similarity: *M* = 0.13, *SD* = 0.23, and Team Interaction distance: *M* = 9.48, *SD* = 3.21). Means within conditions for Task MM similarity are as follows: two-person intact teams (*M* = 0.46, *SD* = 0.25), three-person intact teams (*M* = 0.32, *SD* = 0.20), membership loss teams (*M* = 0.32, *SD* = 0.28), and membership loss with replacement teams (*M* = 0.42, *SD* = 0.23). Means within conditions for Team Interaction MM similarity are as follows: two-person intact teams (*M* = 0.16, *SD* = 0.28), three-person intact teams (*M* = 0.14, *SD* = 0.19), membership loss teams (*M* = 0.14, *SD* = 0.26), and membership loss with replacement teams (*M* = 0.09, *SD* = 0.17). Means within conditions for Task MM distance are as follows: two-person intact teams (*M* = 11.45, *SD* = 4.91), three-person intact teams (*M* = 11.89, *SD* = 2.07), membership loss teams (*M* = 13.15, *SD* = 4.21), and membership loss with replacement teams (*M* = 11.50, *SD* = 4.08). Finally, means within conditions for Team Interaction MM distance are as follows: two-person intact teams (*M* = 8.61, *SD* = 3.28), three-person intact teams (*M* = 10.17, *SD* = 3.49), membership loss teams (*M* = 10.34, *SD* = 3.61), and membership loss with replacement teams (*M* = 8.82, *SD* = 2.18).

Teammate TMMs were calculated using mini-IPIP, a 20-item short form of the International Personality Item Pool-Five-Factor Model measure (Donnellan et al., 2006). Recall that Teammate TMMs include general preferences for working (based on personality), as well as levels of expertise. This particular study was focused on *ad hoc* teams engaging in customer service-related tasks; therefore, the personality dimension of Teammate TMMs was the most appropriate measure, as members would have more opportunity to observe personality characteristics than prior expertise. Prior research on TMMs has included personality identification and similarity as evidence of the Teammate TMMs (e.g., Lim and Klein, 2006). Each member was required to complete this measure about themselves and about every other member of the team. To compute similarity and distance indices, a mean was calculated for each subscale (i.e., openness to experience, conscientiousness, extroversion, agreeableness, and neuroticism) per person. These means were then compared for each dyadic pair within the team (self to other rating of self). These dyadic comparisons were then averaged to create a “team member” average and all team member averages were aggregated, using the mean, to create a teammate similarity SMM index or distance SMM index. These team level variables were used in all analyses. Overall means and standard deviations across conditions for each index are as follows: similarity (*M* = 0.47, *SD* = 0.27) and distance (*M* = 2.25, *SD* = 0.45). Within conditions, means were as follows for the similarity index: two-person intact teams (*M* = 0.56, *SD* = 0.32), three-person intact teams (*M* = 0.50, *SD* = 0.26), three-person membership loss teams (*M* = 0.37, *SD* = 0.26), and three-person membership loss with replacement teams (*M* = 0.44, *SD* = 0.23). For the distance index, means within conditions were as follows: two-person intact teams (*M* = 2.08, *SD* = 0.49), three-person intact teams (*M* = 2.22, *SD* = 0.41), three-person membership loss teams (*M* = 2.31, *SD* = 0.47), and three-person membership loss with replacement teams (*M* = 2.39, *SD* = 0.42).

#### Adaptive Performance

Performance was measured using a card-sorting task. At Time I, participants were given 5 min to place cards listing each patient into the correct triage level. As knowledge about patient problems was distributed among team members (e.g., not all patients needing care were seen in the simulation or listed in patient files), all members needed to work together to successfully categorize all patients. A similar card-sorting task was given for Time II. Adaptive performance was calculated as the difference between Time I and Time II (Time II – Time I). Means for Adaptive Performance within conditions were as follows: two-person intact teams (*M* = 0.67, *SD* = 1.95), three-person intact teams (*M* = 1.87, *SD* = 2.50), three-person membership loss teams (*M* = 1.40, *SD* = 3.23), and three-person membership loss with replacement teams (*M* = 0.13, *SD* = 3.50).

## Results

As expected, there was no significant difference in Time I Performance across the four experimental conditions, *F*(3,56) = 0.68, *p* = 0.57, η^2^ = 0.04, suggesting no spurious differences from random assignment. Descriptive statistics and Pearson product-moment correlations are reported in Table 1. Table 2 contains condition intercorrelations among performance variables.

**TABLE 1:** Intercorrelations, means, and standard deviations for study variables.

	**1**	**2**	**3**	**4**	**5**	**6**	**7**	**8**	**9**	**10**	**11**	**12**	**13**	**14**	**15**
Task TMM Corr.	–														
Team Interaction TMM Corr.	–0.01	–													
Teammate TMM Corr.	0.12	–0.01	–												
Task TMM Euc. Dist.	–0.51^∗∗^	–0.14	–0.34^∗∗^	–											
Team Interaction TMM Euc. Dist.	–0.11	–0.18	−0.28^∗^	0.32	–										
Teammate TMM Euc. Dist.	–0.14	0.07	–0.54^∗∗^	0.17	0.08	–									
Total Info Sharing	–0.01	–0.07	–0.08	–0.02	–0.01	0.30^∗^	–								
GPA (Average for Team)	–0.05	–0.20	0.22	–0.23	–0.26	–0.05	0.13	–							
APGO (Team)	–0.08	0.04	0.08	0.10	–0.02	0.03	0.08	0.05	–						
Team Tolerance for Ambiguity	–0.25	0.10	0.02	–0.01	–0.17	0.003	0.15	0.09	–0.49^∗∗^	–					
Team Familiarity	–0.09	0.09	0.06	–0.03	0.18	0.08	0.09	0.15	0.01	0.12	–				
Role Comprehension	–0.06	0.08	–0.07	–0.04	0.07	0.03	–0.03	–0.08	–0.08	0.09	–0.10	–			
Performance Time I	0.04	0.16	0.19	0.06	–0.17	–0.04	0.12	0.09	–0.001	0.26^∗^	–0.05	–0.11	–		
Performance Time II	–0.002	0.14	0.16	–0.06	–0.16	–0.05	0.000	0.12	0.06	0.18	–0.13	0.07	0.29^∗^	–	
Adaptive Performance	–0.03	–0.01	–0.02	–0.10	0.01	–0.01	–0.10	0.03	0.05	–0.07	–0.07	0.15	–0.58^∗∗^	0.61^∗∗^	–
*M*	0.38	0.13	0.47	12.00	9.48	2.25	9.23	3.23	2.60	3.50	4.44	3.73	4.10	5.12	1.02
*SD*	0.14	0.23	0.27	3.92	3.21	0.45	6.04	0.39	0.53	0.33	8.46	0.43	2.36	2.44	2.87

*^∗^*p* ≤ 0.05; ^∗∗^*p* ≤ 0.01.*

**TABLE 2:** Intercorrelations, means, and standard deviations for performance variables by condition.

	**1**	**2**	**3**
**2-person Intact Teams**			
Performance Time I	–		
Performance Time II	0.62^∗^	–	
Adaptive Performance	−0.62^∗^	0.23	–
*M*	4.40	5.01	0.67
*SD*	2.41	1.95	1.95
**3-person Intact Teams**			
Performance Time I	–		
Performance Time II	0.41	–	
Adaptive Performance	–0.38	0.69^∗∗^	–
*M*	3.93	5.80	1.87
*SD*	1.98	2.54	2.50
**Membership Loss Teams (3 → 2 members)**			
Performance Time I	–		
Performance Time II	0.15	–	
Adaptive Performance	–0.64^∗∗^	0.66^∗∗^	–
*M*	3.47	4.87	1.40
*SD*	2.45	2.50	3.23
**Membership Replacement Teams (3 → 3 members)**			
Performance Time I	–		
Performance Time II	0.18	–	
Adaptive Performance	−0.61^∗^	0.67^∗∗^	–
*M*	4.60	4.73	0.13
*SD*	2.64	2.82	3.50

*^∗^*p* ≤ 0.05; ^∗∗^*p* ≤ 0.01.*

Hypotheses H2 through H4 tested the mediating effects of learning. Although such tests have traditionally been guided by a multistep process proposed by Baron and Kenny (1986), more recent work suggested methodological shortcomings of this approach (e.g., MacKinnon et al., 2002; Edwards and Lambert, 2007). Preacher and Hayes (2004) suggested a different, more powerful, approach called bootstrapping, which can be applied using an SPSS macro (Kolbe et al., 2009). Adaptive performance was regressed onto membership condition, as well as the various TMM measures. Models were tested using correlations and Euclidean distances, run separately, as (a) results can differ based on metrics (Smith-Jentsch, 2009) and (b) there is currently no theory guiding metric selection for adaptive performance.

### Two-Person Intact vs. Membership Loss Teams

#### Similarity Index

H1 suggested that condition would predict performance and H2 suggested that Task TMMs would partially mediate the relationship between membership fluidity (two-person intact teams and membership loss teams) and adaptive team performance. Results did not support mediation for membership loss teams and two-person intact teams when Task TMMs were operationalized using the similarity index (see Table 3) as Task TMMs were not significantly related to condition, β = −0.01, *t*(28) = −0.14, *p* = 0.89, nor were they significant predictors of Performance, β = −0.50, *t*(28) = −0.19, *p* = 0.85. The indirect effect of condition on performance was not in the hypothesized direction (β = 1.05), nor was it significant (*p* = 0.38).

**TABLE 3:** Mediation: TMMs, 2-person intact and membership loss teams.

**Variable**	**β**	***SE***	***t***	***p***	**Confidence Interval**	
					**LL 95% CI**	**UL 95% CI**
**Direct and Total Effects – CORRELATION**						
Adaptive Performance Regressed on Condition^a^	0.33	1.49	0.22	0.83	–2.77	3.42
Task TMMs Regressed on Condition^a^	–0.01	0.10	–0.14	0.89	–0.23	0.20
Team Interaction TMMs Regressed on Condition^a^	–0.09	0.11	–0.78	0.44	–0.31	0.14
**Teammate TMMs Regressed on Condition^a^**	**−0.32**	**0.11**	**−2.86**	**0.01^∗^**	**−0.55**	**−0.09**
Adaptive Performance Regressed on Task TMMs, controlling for Condition^a^	–0.50	2.64	–0.19	0.85	–6.00	5.00
Adaptive Performance Regressed on Team Interaction TMMs, controlling for Condition^a^	–2.29	2.34	–0.98	0.34	–7.16	2.59
Adaptive Performance Regressed on Teammate TMMs, controlling for Condition^a^	–1.65	2.50	–0.66	0.52	–6.84	3.54
Adaptive Performance Regressed on Condition^a^, including TMMs as Mediator (Total Effects Model^b^)	1.05	1.18	0.89	0.38	–1.38	3.49
**Direct and Total Effects – EUCLIDEAN DISTANCE**						
Adaptive Performance Regressed on Condition^a^	1.34	1.52	0.88	0.39	–1.83	4.51
**Task TMMs Regressed on Condition^a^**	**3.21**	**1.89**	**1.70**	**0.10^∗1^**	**−0.69**	**7.11**
**Team Interaction TMMs Regressed on Condition^a^**	**3.86**	**1.19**	**3.24**	**0.004^∗∗^**	**1.40**	**6.31**
Teammate TMMs Regressed on Condition^a^	0.23	0.22	1.09	0.29	–0.21	0.68
Adaptive Performance Regressed on Task TMMs, controlling for Condition^a^	–0.01	0.15	–0.05	0.97	–0.31	0.30
Adaptive Performance Regressed on Team Interaction TMMs, controlling for Condition^a^	–0.09	0.23	–0.37	0.71	–0.56	0.39
Adaptive Performance Regressed on Teammate TMMs, controlling for Condition^a^	0.27	1.29	0.21	0.84	–2.41	2.95
Adaptive Performance Regressed on Condition^a^, including TMMs as Mediator (Total Effects Model^b^)	1.05	1.18	0.89	0.38	–1.38	3.49

*n* = 30 teams. Bootstrap sample size = 5,000. LL, lower limit; CI, confidence interval; UL, upper limit. Condition^*a*^ = Conditions 2 (2-Person Intact Teams) and 4 (Membership Loss Teams). Total Effects Model^*b*^ = Direct Effects + Indirect Effects. Controlling for Average GPA, APGO, Tolerance for Ambiguity, and Role Comprehension. ^*1^*p* = 0.05, one-tailed, ^∗∗^*p* ≤ 0.01.

H3 suggested Team Interaction TMMs would partially mediate the relationship between membership fluidity (two-person intact teams and membership loss teams) and adaptive team performance. These results did not suggest mediation either (Table 3). Team Interaction TMMs were not significantly related to condition, β = −0.09, *t*(28) = −0.78, *p* = 0.44. Furthermore, Team Interaction TMMs were not significant predictors of Performance, β = −2.29, *t*(28) = −0.98, *p* = 0.34.

#### Euclidian Distance Index

However, when using the relative distance metric, the degree of Euclidean distance for Task TMMs was significantly predicted by condition, β = 3.21, *t*(28) = 1.70, *p* = 0.05. Essentially, membership loss teams had greater distance among Task TMMs ratings than two-person intact teams. Similarly, Team Interaction TMMs were significantly predicted by condition, β = 3.86, *t*(28) = 3.24, *p* = 0.004.

### Three-Person Intact vs. Membership Replacement Teams

#### Similarity Index

As reported in Table 4, analyses were conducted to test the mediation hypotheses for three-person intact teams compared to membership replacement teams. When operationalized using the similarity index, neither Task TMMs [β = 0.11, *t*(28) = 1.23, *p* = 0.23] nor Teammate TMMs [β = −0.08, *t*(28) = −0.88, *p* = 0.39] were predicted by condition. However, condition did predict adaptive performance in the hypothesized direction, β = −2.06, *t*(28) = −1.79, *p* = 0.04.

**TABLE 4:** Mediation: TMMs, 3-person intact and membership loss w/replacement teams.

**Variable**	**β**	***SE***	***t***	***P***	**Confidence Interval**	
					**LL 95% CI**	**UL 95% CI**
**Direct and Total Effects – CORRELATION**						
Adaptive Performance Regressed on Condition^a^	–1.77	1.26	–1.41	0.17	–4.37	0.83
Task TMMs Regressed on Condition^a^	0.11	0.09	1.23	0.23	–0.07	0.28
Team Interaction TMMs Regressed on Condition^a^	0.30	0.51	0.51	0.62	–0.19	0.10
Teammate TMMs Regressed on Condition^a^	–0.08	0.09	–0.88	0.39	–0.27	0.11
Adaptive Performance Regressed on Task TMMs, controlling for Condition^a^	–0.55	2.90	–0.19	0.85	–6.56	5.46
Adaptive Performance Regressed on Team Interaction TMMs, controlling for Condition^a^	4.50	3.59	1.25	0.22	–2.95	11.94
Adaptive Performance Regressed on Teammate TMMs, controlling for Condition^a^	0.29	2.62	0.11	0.91	–5.15	5.72
**Adaptive Performance Regressed on Condition^a^, including TMMs as Mediator (Total Effects Model)^b^**	**−2.06**	**1.15**	**−1.79**	**0.09^∗^**	**−4.43**	**0.32**
**Direct and Total Effects – EUCLIDEAN DISTANCE**						
Adaptive Performance Regressed on Condition^a^	–1.77	1.26	–1.41	0.17	–4.37	0.83
Task TMMs Regressed on Condition^a^	–0.39	1.27	–0.31	0.76	–3.02	2.23
Team Interaction TMMs Regressed on Condition^a^	–1.66	1.08	–1.53	0.14	–3.88	0.57
Teammate TMMs Regressed on Condition^a^	0.17	0.16	1.04	0.31	–0.17	0.51
Adaptive Performance Regressed on Task TMMs, controlling for Condition^a^	–0.23	0.19	–1.23	0.23	–0.61	0.16
Adaptive Performance Regressed on Team Interaction TMMs, controlling for Condition^a^	–0.15	0.23	–0.688	0.50	–0.62	0.31
Adaptive Performance Regressed on Teammate TMMs, controlling for Condition^a^	–0.12	1.48	–0.08	0.93	–3.19	2.94
**Adaptive Performance Regressed on Condition^a^, including TMMs as Mediator (Total Effects Model)**	**−2.06**	**1.15**	**−1.79**	**0.09^∗^**	**−4.43**	**0.32**

**n* = 30 teams. Bootstrap sample size = 5,000. LL, lower limit; CI, confidence interval; UL, upper limit. Condition^*a*^ = Conditions 3 (3-Person Intact Teams) and 5 (Membership Loss w/Replacement Teams), Total Effects Model^*b*^ = Direct Effects + Indirect Effects. Controlling for Average GPA, Team Familiarity, and Role Comprehension. ^∗^*p* = 0.04 level, one-tailed.*

#### Euclidian Distance Index

Results for the relative distance TMM metric also did not support mediation for Task or Teammate TMMs. Task TMMs, operationalized as Euclidean distance, were not significantly predicted by condition, β = −0.39, *t*(28) = −0.31, *p* = 0.76. Condition also did not predict Teammate TMMs, β = 0.17, *t*(28) = 1.04, *p* = 0.14 with the distance metric. Further, neither of the TMMs distance indices predicted Adaptive Team Performance [Task:β = −0.23, *t*(28) = −1.23, *p* = 0.23; Teammate:β = −0.12, *t*(28) = −0.08, *p* = 0.93].

### Exploratory Analyses

Upon reflection, the task likely determined the extent to which members were able to gain information regarding member preferences/tendencies. The task in this study was social in nature, comprised of *ad hoc* teams. So, skewness and kurtosis analyses were conducted across conditions. Results suggest that familiarity data were not normally distributed. Specifically, the positive skewness value (2.57) suggests that the majority of the responses were less than the mean while the kurtosis level (6.79) suggests that the data are more closely clustered around the mean (i.e., low lower levels of data fluctuation than what is seen in normal distributions). Together, this suggests that participants generally had low levels of familiarity with one another. As such, members could only develop similar views of easily observed characteristics, which could have led to spurious ratings of unobserved personality traits (e.g., without any demonstration of cues for openness to experience, members would have little insight into that personality factor). The use of an aggregated Teammate TMM (i.e., aggregation of all five personality factors) could have, therefore, led to attenuated correlations or inflated Euclidean distances, limiting explanatory power. Thus, teammate TMM was re-operationalized at the factor level (separate personality constructs) and additional analyses were then conducted using these separate variables.

The *Agreeableness* factor was predicted by condition, β = −0.14, *t*(28) = −2.23, *p* = 0.04 (see Table 5). Essentially, intact teams had more similar Teammate TMMs regarding members’ levels of agreeableness than did membership loss with replacement teams. Also, the *Neuroticism* factor significantly predict adaptive performance, β = 4.49, *t*(28) = 1.96, *p* = 0.03. Teams that correctly identified fellow members’ levels of neuroticism performed better at Time II than Time I. The Neuroticism factor (Euclidean distance) was predicted by condition [β = −0.43, *t*(28) = −1.69, *p* = 0.05]. Additionally, the Agreeableness factor, operationalized as Euclidean distance [β = −3.57, *t*(28) = −2.90, *p* = 0.01], significantly predicted adaptive team performance. Teams who had more similar TMMs regarding members’ levels of agreeableness performed better at Time II than at Time I. Interestingly, when considered along with the factors of Teammate TMMs, Task TMMs significantly predicted adaptive team performance [β = −0.30, *t*(28) = −1.72, *p* = 0.05].

**TABLE 5:** Mediation: teammate TMM dimensions—correlations, exploratory analyses.

**Variable**	**β**	***SE***	***T***	***P***	**Confidence Interval**	
					**LL 95% CI**	**UL 95% CI**
**Direct and Total Effects – CORRELATION**						
Adaptive Performance Regressed on Condition^a^	–1.76	1.47	–1.20	0.25	–4.82	1.30
Task TMMs Regressed on Condition^a^	0.05	0.08	0.60	0.55	–0.12	0.23
Team Inter. TMMs Regressed on Condition^a^	–0.07	0.07	–0.99	0.33	–0.22	0.08
Teammate O TMMs Regressed on Condition^a^	0.01	0.06	–0.09	0.93	–0.13	0.12
**Teammate C TMMs Regressed on Condition^a^**	**0.19**	**0.09**	**2.08**	**0.05**	**0.002**	**0.38**
Teammate E TMMs Regressed on Condition^a^	–0.07	0.11	–0.60	0.55	–0.30	0.17
**Teammate A TMMs Regressed on Condition^a^**	**−0.14**	**0.06**	**−2.23**	**0.04**	**−0.27**	**−0.01**
Teammate N TMMs Regressed on Condition^a^	0.09	0.11	0.83	0.42	–0.14	0.32
Adaptive Performance Regressed on Task TMMs, controlling for Condition^a^	1.56	2.95	0.53	0.60	–4.57	7.69
Adaptive Performance Regressed on Team Interaction TMMs, controlling for Condition^a^	2.39	3.95	0.61	0.55	–5.82	10.61
Adaptive Performance Regressed on Teammate O TMMs, controlling for Condition^a^	–3.95	4.20	–0.94	0.36	–12.69	4.78
Adaptive Performance Regressed on Teammate C TMMs, controlling for Condition^a^	–4.35	3.20	–1.36	0.19	–11.01	2.31
Adaptive Performance Regressed on Teammate E TMMs, controlling for Condition^a^	–2.02	2.11	–0.96	0.35	–6.41	2.37
Adaptive Performance Regressed on Teammate A TMMs, controlling for Condition^a^	–3.38	4.38	–0.77	0.45	–12.49	5.73
**Adaptive Performance Regressed on Teammate N TMMs, controlling for Condition^a^**	**4.49**	**2.29**	**1.96**	**0.06^∗^**	**−0.27**	**9.26**
Adaptive Performance Regressed on Condition^a^, including TMMs as Mediator (Total Effects Model)^b^	–1.73	1.11	–1.56	0.13	–4.01	0.54
**Direct and Total Effects – EUCLIDEAN DISTANCE**						
Adaptive Performance Regressed on Condition^a^	–1.05	1.17	–0.90	0.38	–3.49	1.39
Task TMMs Regressed on Condition^a^	0.48	1.24	0.39	0.70	–2.07	3.03
Team Inter. TMMs Regressed on Condition^a^	–1.71	1.13	–1.51	0.14	–4.05	0.63
Teammate O TMMs Regressed on Condition^a^	–0.29	0.20	–1.48	0.15	–0.70	0.11
Teammate C TMMs Regressed on Condition^a^	0.20	0.19	1.06	0.30	–0.19	0.60
Teammate E TMMs Regressed on Condition^a^	0.01	0.20	0.03	0.98	–0.40	0.41
Teammate A TMMs Regressed on Condition^a^	0.08	0.24	0.35	0.73	–0.40	0.57
**Teammate N TMMs Regressed on Condition^a^**	**−0.43**	**0.26**	**−1.69**	**0.10^∗^**	**−0.96**	**0.09**
**Adaptive Performance Regressed on Task TMMs, controlling for Condition^a^**	**−0.30**	**0.18**	**−1.72**	**0.10^∗^**	**−0.67**	**0.06**
Adaptive Performance Regressed on Team Interaction TMMs, controlling for Condition^a^	–0.19	0.18	–1.08	0.30	–0.57	0.18
**Adaptive Performance Regressed on Teammate O TMMs, controlling for Condition^a^**	**3.94**	**1.46**	**2.69**	**0.01**	**0.89**	**6.98**
Adaptive Performance Regressed on Teammate C TMMs, controlling for Condition^a^	0.37	1.33	0.28	0.79	–2.40	3.13
Adaptive Performance Regressed on Teammate E TMMs, controlling for Condition^a^	–0.90	1.26	–0.72	0.48	–3.51	1.71
**Adaptive Performance Regressed on Teammate A TMMs, controlling for Condition^a^**	**−3.57**	**1.23**	**−2.90**	**0.01**	**−6.14**	**−1.01**
Adaptive Performance Regressed on Teammate N TMMs, controlling for Condition^a^	–1.04	0.91	–1.14	0.27	–2.93	0.86
Adaptive Performance Regressed on Condition^a^, including TMMs as Mediator (Total Effects Model)^b^	–1.73	1.11	–1.56	0.13	–4.01	0.54

**n* = 30 teams. Bootstrap sample size = 5,000. LL, lower limit; CI, confidence interval; UL, upper limit. Condition^*a*^ = Conditions 3 (3-Person Intact Teams) and 5 (Membership Loss w/Replacement). Total Effects Model^*b*^ = Direct Effects + Indirect Effects. Controlling for Average GPA, APGO, and Team Familiarity. ^∗^*p* = 0.03, one-tailed (finding is in hypothesized direction).*

## Discussion

The hypotheses in this study essentially described a mediation model, derived from theory, to explain one possible mechanism that enables teams to adapt: TMMs. It was hypothesized that teams in the experimental conditions would not develop the same level of sharedness in mental models as teams who did not experience any membership changes. Membership fluidity was expected to negatively influence adaptive performance but that relationship was predicted to be partially mediated by the lack of sharedness in mental models. Although results did not support partial mediation, three-person intact teams demonstrated greater adaptive performance than teams who experienced membership loss with replacement. Furthermore, two-person intact teams developed more similar task and team interaction TMMs than teams who lost a member when TMMs were indexed as a Euclidean distance score. Contrary to predictions, there were no differences in the level of sharedness regarding Task or Teammate TMMs for three-person intact teams as compared to membership loss with replacement teams.

**FIGURE 3:**
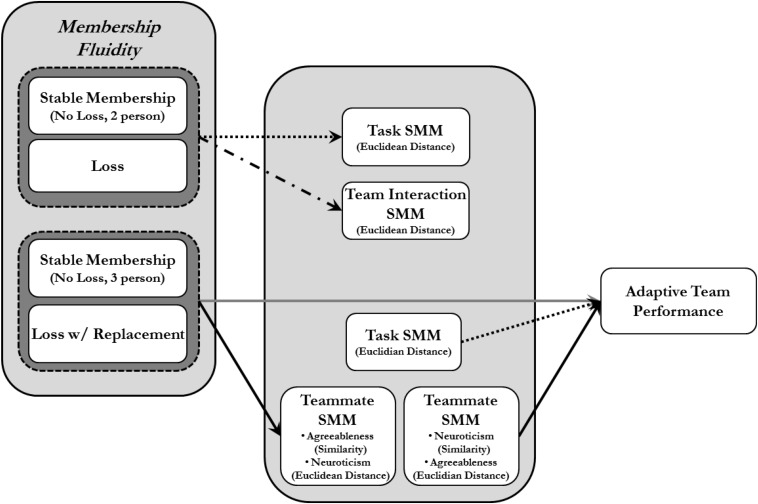
Actual relationship among hypothesized study variables.

When Teammate TMMs were operationalized as individual personality factors (i.e., the Big 5 – openness to experience, conscientiousness, extroversion, agreeableness, and neuroticism), three-person intact teams did develop more similar TMMs regarding the agreeableness factor (similarity index) and the neuroticism factor (distance index) than membership loss with replacement teams. Additionally, when operationalized as Euclidean distance, the Agreeableness factor significantly predicted adaptive team performance—specifically, the smaller the distance (i.e., more similar the TMMs), the greater the adaptive performance in teams. When operationalized as the similarity index, the neuroticism factor significantly predicted adaptive team performance as well, such that the more similar the TMMs, the greater the adaptive performance in teams. Finally, when factors were included in the analyses, Task TMMs significantly predicted adaptive team performance (distance index). Figure 3 shows a model of the supported relationships.

### Theoretical and Practical Implications

Theoretically, this research extends our current understanding of team adaptation by moving beyond a change in task complexity or one type of change in team configuration to investigate team member loss as well as team member loss with replacement. This may more accurately represent the dynamic flow of individuals among teams that is common in organizations today. Team research is just beginning to consider membership fluidity as a potential issue in process loss as early work on team adaptation with regard to membership change has largely been theoretical (Summers et al., 2012). Providing empirical evidence regarding the influence of fluidity on TMM sharedness helps move the field forward in terms of synthesizing existing assumptions into meaningful theory.

Results support a direct negative influence of membership loss with replacement on adaptive team performance, which is consistent with previous research on team familiarity (Goodman and Leyden, 1991; Smith-Jentsch et al., 2009). Although results did not support TMMs mediating the relationship between the various condition and performance in this study, membership fluidity did negatively influence the development of task, team interaction, and teammate TMMs, depending on whether teams experienced membership loss or change. However, there were inconsistent findings with regard to the relationship of these variables to adaptive team performance, depending on operationalization and condition. This may be due to the fact that TMMs do not exert a direct effect on adaptive performance, but rather an indirect effect through team process (e.g., Mathieu et al., 2000) or an interaction of TMMs (Smith-Jentsch et al., 2005). Thus, theory must link specific types of TMMs (rather than overall shared cognition constructs) to particular team processes to drive future research (Smith-Jentsch, 2009).

Although none of the hypothesized TMMs influenced adaptive performance, when operationalized at the factor level, teammate (agreeableness, neuroticism) and task TMMs significantly predicted adaptive team performance. Research within the team domain rarely considers multiple types of TMMs within a single study, especially since Mathieu et al. (2000) suggested that the four types of TMMs outlined by Cannon-Bowers et al. (1993) ultimately depict two major content domains. A review of the team literature noted that few studies have conceptualized more than one dimension of TMMs (Mathieu et al., 2008). When more than one dimension has been studied, researchers almost unanimously focus on task and team TMMs, ignoring *teammate* TMMs and instead focusing on *team interaction* TMMs. Other than the work from Smith-Jentsch et al. (2001, 2009), the majority of research that has considered the degree to which team member preferences are known, has typically resided in the transactive memory system literature. Transactive memory systems are considered to be the collection of individually held information and the knowledge regarding the distribution of that information among team members (Wegner, 1986) and some would argue, includes the degree to which members hold knowledge of other member work preferences (e.g., Lewis et al., 2007). In fact, results are consistent (i.e., differences in TMS between intact and reconstituted teams) with such findings. Indeed, in this study, intact teams had significantly higher levels of all three types of TMMs measured (i.e., task, team interaction, and teammate). However, findings differed based on whether teams lost or changed members.

Furthermore, findings from the exploratory analyses suggest that multiple dimensions of TMMs—particularly teammate—differentially influence results. This particular task was a customer service task, and the hospital staff and patients were scripted specifically to be challenging to work with, providing many opportunities for teammates to observe levels of agreeableness. Consider the member who is interacting with the simulation (Waiting Room Staffer) who specifically sees all patients and hospital staffers, some of whom are difficult to deal with. It is very easy to determine one’s level of agreeableness when observing someone interacting with the simulation. During the second action phase, members could have leveraged such information to alter how they interacted with that person (be more candid for highly agreeable individuals and be more patient with those lower on agreeableness). This change in how members approach their teammates helps everyone gain additional information and thus, could improve performance.

Additionally, the performance measures were timed and a performance reward was offered for the highest-ranking teams. Therefore, the measures focused on both speed and accuracy. This provides many opportunities to observe levels of neuroticism as well. During the next performance episode, effective team members who noticed more neurotic levels of behavior from a teammate during the timed performance measure at Time 1 could elicit information from that person first, to avoid having him/her get flustered toward the end of the time period or perseverate over the information while waiting to contribute, resulting in a member who had confused the details and thus, could negatively influence team performance.

Thus, adaptation theory should discuss how *specific types* of TMMs (and corresponding dimensions) influence adaptation. The Burke et al. (2006) specifically discusses cognitions, suggesting that adaptive team performance, by definition, requires a change in “cognitive or behavioral goal-directed actions or structures to meet expected or unexpected demands” (p. 1192); however, the discussion is limited to generic TMMs, not specifying which types are most important at any given time. Kozlowski et al. (1999) also suggest adaptive performance is comprised of a series of stages, but do not specifically mention shared mental models. However, when considered closely, the underlying mechanisms required for successfully moving through the phases are cognitively based. For example, socialization—the first phase—is focused on reducing social ambiguity, which is often inherent at team formation by seeking knowledge regarding the team. One particular type of knowledge that the authors suggest aids in the socialization process is *interpersonal knowledge*, which is the information that comprises teammate TMMs. Kozlowski also suggests that team orientation aids adaptive performance. The development of a *team orientation* involves the identification of team goals (i.e., what the team is trying to do), team climate (i.e., what it is like to be part of this particular team), and norms for interaction (i.e., acceptable behavior within the team). This provides the necessary boundary conditions within which the team will operate, enabling members to see how each particular individual role aligns with the overall mission of the team and provides a basis for development of shared perceptions (Nieva et al., 1978). This, essentially, describes team interaction TMMs. If adaptation theory can integrate with team cognition theory, there will be greater specificity with regard to the team level cognitions required for effective adaptation, allowing researchers to target specific dimensions of task, team interaction, and teammate TMMs when conducting team adaptation research. Such integration can streamline research efforts, which facilitates translation of science to practice.

As researchers continue to call for more complex investigations into team adaptation phenomena (e.g., Baard et al., 2014; Waller et al., 2016) more theory is needed to guide such efforts. Zajac et al. (2014) attempted to add some clarity to the cognitive domain of adaptive team performance with their theory, integrating TMS and TMMs specifically with adaptive performance, resulting in a model that highlights how TMS and TMMs evolve over time. Indeed researchers (Uitdewilligen et al., 2013) found that mental model updating is positively related to postchange team performance. Thus, future research should incorporate multiple measures of TMMs and include in regression analyses that look at sequential mediators as the timing of the TMM measurement may influence results if only measured once. Further, theory must begin to incorporate time into models of adaptation (Cronin et al., 2011; Kozlowski and Chao, 2012; Waller et al., 2016). Rosen et al. (2011) have outlined a number of principles that should be considered when studying team adaptation with suggested measurement strategies for each principle. Such work can aid researchers in identifying variables and measurement strategies for more complex investigations.

On a more practical level, organizations trying to recover from economic hardships are tightening control over expenditures by redistributing workload among existing employees rather than hiring additional help. Thus, experienced workers are often removed from one team and placed on another team. Although much adaptive team performance research has focused on integration of a new member (e.g., Moreland and Levine, 2001), research has not adequately considered fluid team configurations (Summers et al., 2012; Tannenbaum et al., 2012).

This research provides a necessary first step toward understanding the implications of both membership loss and membership loss with replacement on adaptive team performance. Various membership fluidity conditions differentially influenced the sharedness of TMMs. Essentially, removing members without replacement in decision-making tasks requiring pooled, uniquely held knowledge caused decrements to the sharedness of TMMs (task and team interaction). Replacing lost teammates with members who were familiar with the task did not result in decrements to task TMMs; however, it did influence the sharedness of teammate TMMs. Ultimately, task and teammate TMMs directly influenced adaptive performance when operationalized as personality factors. These findings suggest organizations relying upon such teams cannot engage in downsizing or team reconfigurations without incurring some degree of process loss—and potentially, performance decrements. Thus, organizations should focus on knowledge management to store task-relevant information so it remains easily accessible to teams. Organizations should also encourage teams to take time to engage in interpersonal knowledge sharing and role specification discussions (Kozlowski et al., 1999; Burke et al., 2006) to provide mechanisms for developing a shared understanding of the task(s) and the team.

### Limitations and Future Research

Hypothesis testing did not fully support the supposition that high shared task, team interaction and teammate TMMs would alleviate the negative effects of membership fluidity on performance. The team mental model literature emphasizes overlapping knowledge of team members as a critical predictor of team effectiveness (Cannon-Bowers et al., 1993; Mathieu et al., 2000). However, researchers have suggested that shared knowledge encompasses perspectives that are both shared and complementary and further argue that complementary perspectives are most appropriate for heterogeneous teams with distinct roles where performance relies on uniquely held knowledge (Cooke et al., 2000, 2003)—similar to the notion of transactive memory. In fact, Cooke et al. (2000) have suggested that in such teams, researchers should use knowledge distribution metrics to identify where specific knowledge lies as gaps can be compensated for if that knowledge is held by other members. In teams requiring pooling of uniquely held knowledge, measuring overlapping knowledge may not be predictive of what is truly required for successful performance (Mohammed and Dumville, 2001), particularly adaptation. Adaptation theory should, thus, incorporate such knowledge to spur future research.

The decision to remove the Claims Staffer could have influenced results. It was speculated that this particular role required uniquely held knowledge required for effective performance (critical updates provided by the experimenter). Removal of the Waiting Room Staffer, who interacted directly with the simulation, may have led to different results. Team members had much greater opportunities to observe personality factors based on tasks requirements of this role. Perhaps through removal of this member, condition would have more strongly predicted overall Teammate TMMs and such TMMs would have been related to adaptive performance because the Waiting Room Staffer had more detailed patient knowledge. Removal of this member would have necessitated reconfiguration, as someone would have been required to change roles to engage with the simulation, thus, impacting team interaction TMMs. Finally, this particular role was qualitatively different from the Claims or Records Staffer. Removal of the Waiting Room Staffer would have required remaining members in the loss condition to develop an understanding of a different task, perhaps influencing sharedness of task mental models. Future research should investigate results based on different role removals.

As noted previously, Euclidean distance scores were found to be significant more often than correlation scores. Finally, some SMM findings were associated with the similarity index, while others were based on the Euclidean distance. Practically speaking, it is important to consider measurement indices and this study adds additional support to the notion that measurement matters. Smith-Jentsch (2009) articulated these issues in her chapter on team cognitions. She noted that different metrics produce different results and careful consideration should be placed on the specific research questions to select the most appropriate metric. Resick et al. (2010) added additional support to Smith-Jentsch’s argument by empirically demonstrating that different SMM elicitation methods result in varied relationships with outcomes of interest, such as adaptive team performance. This study is yet another indicator of the importance of measurement. SMM correlations (i.e., similarity indices) were more predictive at times, however, the Euclidian distance scores provided more overall support for hypothesis (and exploratory analysis) testing. This is possibly due to the fact that correlations can be attenuated when members completely agree (restriction of range), either through item or aggregate team-level analyses (i.e., an average self-rating of 4 across items compared to an average other rating of 4 results in lack of a correlation or a correlation of 0.0). However, if the pattern of responses were different such that one rating was 4-5-3 and the other rating was 3-5-4, the distance score would reflect an actual Euclidean distance score of 1.0, which indicates high levels of agreement. Similarly, correlation ratings can also be inflated, in the case of a “perfect” correlation based on the same pattern of responses, but different actual ratings. Consider one person rating 4-5-4-4 and another rating 2-3-2-2. This would be considered a perfect correlation of 1.0. Yet, when calculated as the distance score, it is 4.0, which is considerably less “agreement” than indicated by a perfect correlation. Essentially, the correlations measure the how similar members were able to rate patterns of responses, whereas Euclidean distances measure absolute distance among ratings (whether members figure out that others were either high or low, but just were slightly off regarding the specific pattern of responses). In cases with restriction of range (as discussed above), the Euclidean distance score would more accurately capture the true nature of relatedness. Yet caution must be taken when considering results using distance score metrics. Although it is true that distance scores may yield attenuated relationships, some argue that they are problematic as they are generally unreliable and polynomial regression should be used instead (which generally requires a large sample size); thus, future research should consider collecting more samples and running analyses with polynomial regression (Edwards, 2001).

The nature of the tasks within this study forced members to engage in independent taskwork, and then suddenly shift to interdependent teamwork. Research should consider how such transitions influences the development of TMMs and adaptive performance as previous research suggests that teams have more performance problems when shifting from a functional structure to a divisional structure (Moon et al., 2004). Thus, there could be different performance implications when shifting from interdependent to independent as compared to the independent-interdependent entrainment shifts experienced by teams in this effort.

## Conclusion

To provide practitioners with evidence-based guidelines for training teams to be adaptive to changing conditions (e.g., membership changes), conceptual direction is required and, more importantly, empirical evidence stemming from rigorous theoretical tests. Based upon these results, it is argued that team adaptation theory, which includes cognitive components, must go deeper than suggesting that overall cognition—or even the general construct of TMMs—is necessary. In particular, there must be integration of empirical findings regarding specific aspects of cognition to begin to theorize relationships among key constructs, especially in teams with fluid membership as they are more and more common in environments across work domains. Research that considers membership fluidity, such as this effort, can help shed light into the nature of such required theoretical changes necessary to effectively guide future research efforts. Such work is critical to move the field forward in a meaningful manner and really explore how the cognitive component of teamwork influences team performance in fluid teams.

## Ethics Statement

This study was carried out in accordance with the recommendations of the University of Central Florida Institutional Review Board (UCF IRB). Given that a signed informed consent would be the only identifying information tied to participation, signed informed consent was waived. The protocol was approved by the UCF IRB.

## Author Contributions

The author confirms being the sole contributor of this work and has approved it for publication.

## Conflict of Interest

The authors declare that the research was conducted in the absence of any commercial or financial relationships that could be construed as a potential conflict of interest.
